# Hospital healthcare utilizers with medical and psychiatric claims in the Netherlands: a nationwide study

**DOI:** 10.1186/s12913-022-07798-6

**Published:** 2022-04-11

**Authors:** Maarten van Schijndel, Luc Jansen, Jan Busschbach, Jeroen van Waarde, Andre Wierdsma, Henning Tiemeier

**Affiliations:** 1 Department of Psychiatry, Rijnstate Hospital, Arnhem, the Netherlands; 2 Department of Psychiatry, Erasmus Medical Centre, Epidemiological and Social Psychiatric Research Institute, Rotterdam, the Netherlands; 3 Department of Psychiatry, Section Medical Psychology and Psychotherapy, Erasmus MC, Rotterdam, the Netherlands; 4 Department of Child and Adolescent Psychiatry/Psychology, Erasmus University Medical Center Rotterdam, Rotterdam, The Netherlands; 5 Department of Social and Behavioural Science, Harvard TH Chan School of Public Health, Boston, MA USA

**Keywords:** Psychiatric and medical multimorbidity, Healthcare utilization, Hospital claims

## Abstract

**Background:**

Psychiatric and medical multimorbidity is associated with low quality of life, poor functioning and excess mortality. Differences in healthcare utilization between those receiving co-occurring medical and psychiatric healthcare (HUMPCs) and those only receiving medical (HUMCs) or only psychiatric healthcare (HUPCs) may indicate differences in care accessibility, help-seeking behavior and the risk patterns of medical illness. We aimed to assess the occurrence of psychiatric diagnostic groups in HUMPCs compared to HUPCs and to compare their medical and psychiatric claims expenditures.

**Methods:**

Using Dutch claims data covering psychiatric and medical hospital care in 2010–2011, healthcare utilization differences between HUMPCs and HUPCs were expressed as differences and ratios, accounting for differences in age and sex between groups. Median claims expenditures were then compared between HUMPCs and HUPCs.

**Results:**

HUMPCs had 40% higher median medical cost of claims compared to HUMCs and a 10% increased number of psychiatric claims compared to HUPCs. HUMPCs were more often diagnosed with: organic disorders; behavioral syndromes associated with physiological disturbances and physical factors; mood [affective] disorders; neurotic, stress related and somatoform disorders; and disorders of adult personality and behavior. By contrast, disorders of psychological development, schizophrenia, schizotypal and delusional disorders, behavioral and emotional disorders with usual onset occurring in childhood, and mental and behavioral disorders due to psychoactive substance abuse were less often diagnosed in this group.

**Conclusions:**

Both medical and psychiatric disease become more costly where both are present. For HUMPCs the costs of both medical and psychiatric claims for almost all diagnostic groups were higher than for HUPCs and HUMCs.

## Introduction

Most healthcare systems use delivery practices that separate psychiatric and medical treatment [1]. This division is noteworthy, given the close relationship between psychiatric and medical morbidity. Patients with psychiatric disorders are more likely to suffer from medical illness than the general population [2]. Although all psychiatric patient groups show a high prevalence of medical disorders/problems, patients with schizophrenia spectrum disorders, bipolar disorders, and substance abuse are most at risk for comorbid medical illness [3, 4]. Patients with psychiatric and medical illnesses experience loss of independence, an inferior quality of life, and disproportional increases in care utilization and costs [1, 5–8]. In addition, medical comorbidity accounts for most of the excess mortality in psychiatric patients [7, 9–12]. Deficiencies in the quality of medical care for these patients add to this excess: a mismatch of health preferences and care arrangements [13], reduced accessibility due to stigma [13–15], and difficulties in managing patients’ care avoidance and challenging behaviors [16].

Healthcare utilization studies comparing psychiatric patients with and without medical morbidity indicate: (i) differences in the accessibility of care [14, 17], (ii) differences in help-seeking behavior [18], and (iii) differences in the risk of developing a medical illness [19]. These studies look at specific patient groups. However, nationwide studies comparing hospital care utilization and claims data for psychiatric patients with and without co-occurring medical care are lacking. A nationwide study that identifies utilization differences could help to identify the under-served patient groups and the high care utilizers. Moreover, insight into healthcare utilization patterns could support targeted interventions to improve accessibility and (health-economic) outcomes in the future.

This present study used nationwide health insurance claims data for medical and psychiatric hospital care in the Netherlands. The dataset covered all Dutch inhabitants since healthcare insurance is compulsory. We aimed: (i) to assess if certain psychiatric diagnostic groups occurred more often in healthcare utilizers with medical and psychiatric claims (HUMPCs) than in healthcare utilizers with psychiatric claims (HUPCs) only, and (ii) to compare the medical and psychiatric claims expenditures for HUMPCs to those of HUPCs and healthcare utilizers with medical claims (HUMCs).

We expected to find an over-representation of severe mental illness and substance abuse patients in HUMPCs compared to HUPCs. These patients have a higher risk for medical conditions than the general population [3, 4]. Furthermore, on average, we expected that claims of HUMPCs are higher than the claims of HUPCs or HUMCs.

## Methods

### Data-collection

The Dutch Healthcare Authority (‘Nederlandse Zorgautoriteit’) provided claims data for psychiatric and medical (both inpatient and outpatient) hospital care in 2010–2011, which comprised the total number of claims for Dutch specialist care in these years. In the Netherlands, separate funding systems are in place to reimburse both types of care and comprise a case-mix system using a ‘Diagnosis Related Groups’ system, called ‘DBCs’ [20]. The claims data were anonymized, and this study did not intervene in human subjects. Hence, Dutch law did not require the approval of the Medical Research Ethics Committee and we followed all the necessary guidelines laid by Dutch law (7:457 lid 1 BW, https://english.ccmo.nl/investigators/legal-framework-for-medical-scientific-research/your-research-is-it-subject-to-the-wmo-or-not).

### Selection of patients

**Fig. 1:**
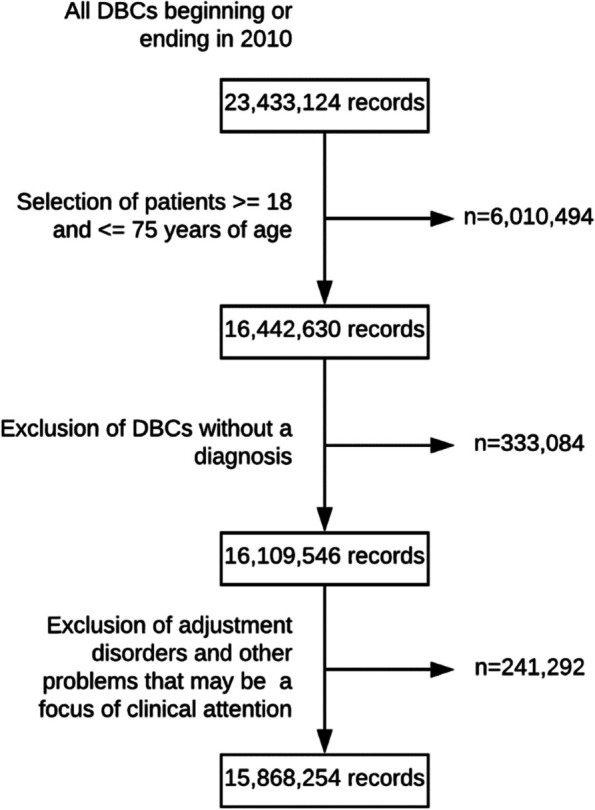
Selection of records in the database

In 2010, the Netherlands had 16.6 million inhabitants with mandatory health insurance. Records from psychiatric and medical hospital care were linked, based on anonymized social security numbers. To ensure reliable linkage, birth year, sex, and residential district codes were also accessed. Figure 1 depicts the selection of patients for whom treatment began or ended in 2010. Patients below 18 and above 75 years of age were excluded because in the Netherlands psychiatric care is financed separately for patients < 18 years, and for the elderly, if cared for in long-term care facilities. Also excluded were psychiatric cases without diagnoses and non-fundable care for ‘adjustment disorders’, and ‘other problems that may be a focus of clinical attention’. The resulting database comprised 5,133,295 unique patients (*n* = 4,532,250 in medical care; *n* = 601,045 in psychiatric care). These were inpatients and outpatients, treated in Dutch specialist care, comprising all general and mental health hospitals and outpatient clinics.

### Care utilization and claims expenditures

Both psychiatric and general medical diagnoses were obtained directly from DBC data. Medical DBCs employed a description of the medical problem for coding based on the International Classification of Disease (ICD-10 [21]), while psychiatric DBCs employed the Diagnostic Statistical Manual of Mental Disorders-IV (DSM-IV-TR) classification [22]. In order to use one classification, the standardized and validated Dutch Healthcare Authority translation table was employed to convert all the DBC data according to the ICD-10. Individual healthcare utilization was assessed by counting psychiatric and medical diagnoses in the study period. If an individual showed more than one (psychiatric or medical) DBC with the same diagnosis, the diagnosis was deemed to be present only once. Then healthcare utilizers who only used medical healthcare were classified as ‘healthcare utilizers with medical claims’ (HUMC). Similarly, healthcare utilizers who only used psychiatric healthcare were classified as ‘healthcare utilizers with psychiatric claims’ (HUPC). Finally, healthcare utilizers with co-occurring medical and psychiatric healthcare utilization were classified as ‘healthcare utilizers with medical and psychiatric claims’ (HUMPC).

### Data analysis

The Proportional Occurrence Difference (POD) and Proportional Occurrence Ratio (POR) were employed to express healthcare utilization differences between the groups just specified. The POD and POR were calculated using the following formulae:

$$\mathrm{POD}=\left(\mathrm{number}\ \mathrm{of}\ \mathrm{HUMPCs}\ \mathrm{in}\ \mathrm{sub}-\mathrm{chapter}/\mathrm{total}\ \mathrm{number}\ \mathrm{of}\ \mathrm{HUMPCs}\right)-\left(\mathrm{HUPCs}\ \mathrm{in}\ \mathrm{psychiatric}\ \mathrm{sub}-\mathrm{chapter}/\mathrm{total}\ \mathrm{number}\ \mathrm{of}\ \mathrm{HUPCs}\right)$$

$$\mathrm{POR}=\mathrm{proportion}\ \mathrm{of}\ \mathrm{HUMPCs}\ \mathrm{in}\ \mathrm{psychiatric}\ \mathrm{sub}-\mathrm{chapter}/\mathrm{proportion}\ \mathrm{of}\ \mathrm{HUPCs}\ \mathrm{in}\ \mathrm{psychiatric}\ \mathrm{sub}-\mathrm{chapter}$$

A POR of > 1 indicated the higher proportional occurrence or over-representation of a psychiatric sub-chapter in HUMPCs compared with its occurrence in HUPCs, while a POR of < 1 indicated the under-representation of the specific subchapter.

In health services research, the standardization of rates is often based on direct or indirect arithmetic methods. When individual data are available, logistic regression models can be used to calculate adjusted rates [23]. The dependent variable in the logistic regression analysis was comorbidity (1 = present; 0 = absent), with age (continuous), sex, and the interaction of age and sex as independent variables. Sensitivity analyses were performed for some important diagnostic groups, since this resulted in minor differences only crude values are reported.

## Results

In our study of 2010 data from the Netherlands, 601,045 individuals claimed specialist psychiatric healthcare and 4,532,404 individuals claimed specialist medical healthcare, characteristics of the included patients are shown in Table 1. Psychiatric patients represented 5% of the Dutch population aged 18–75 years in 2010, while medical patients represented 38%. These figures were in line with official reports on healthcare utilization in the Netherlands in 2012 which showed that 551,000 patients between 18 and 65 were treated in specialist psychiatric care [24]. For all ages, 8.28 million patients visited a Dutch medical hospital. These were all provider contacts and not unique patients [24, 25]. Moreover, the period prevalence of mental health disorders in psychiatric hospital care was congruent with epidemiological data [26].

**Table 1:** Characteristics of included patients

	Psychiatric	Medical
Male	279,709 (46%)	NB
Age		
18–24	83,919 (14%)	394,179 (9%)
25–29	61,396 (10%)	301,628 (7%)
30–34	63,380 (11%)	334,378 (7%)
35–39	69,234 (12%)	372,028 (8%)
40–44	73,127 (12%)	414,541 (9%)
45–49	71,070 (12%)	439,020 (10%)
50–54	60,183 (10%)	449,935 (10%)
55–59	45,839 (8%)	458,509 (10%)
60–64	31,780 (5%)	510,129 (11%)
65–70	20,132 (3%)	418,818 (9%)
70–75	20,985 (3%)	439,085 (10%)
Total	601,045 (100%)	4,532,250 (100%)

Patients aged 18 through 75, treated in Dutch specialist psychiatric care in 2010. Diagnoses are broken down by ICD-10 blocks

### Healthcare utilization

Table 2 shows psychiatric healthcare use broken down for men and women. ‘Mood [affective] disorders’ (27.8%) and ‘neurotic, stress-related and somatoform disorders’ (20.4%) were the most frequent diagnoses in psychiatric care. Women were more often than men diagnosed with ‘behavioral syndromes associated with physiological disturbances and physical factors’ (including eating disorders), ‘neurotic, stress-related and somatoform disorders’, mood [affective] disorders’, and ‘personality disorders’. Men were more often diagnosed with ‘disorders of psychological development’ including autism, ‘substance abuse disorders’, ‘schizophrenia, schizotypal and delusional disorders’ and ‘behavioral and emotional disorders with onset usually occurring in childhood and adolescence’ including attention deficit hyperactivity disorder.

**Table 2:** Psychiatric healthcare utilization

ICD code	ICD subchapter title	All patients		Male patients		Female patients	
		***n***	***%***	***n***	***%***	***n***	***%***
**F00-F09**	**Organic, including symptomatic, mental disorders**	14,724	2.4%	8023	2.9%	6701	2.1%
**F10-F19**	**Mental and behavioral disorders due to psychoactive substance abuse**	67,654	11.3%	50,911	18.2%	16,743	5.2%
**F20-F29**	**Schizophrenia, schizotypal and delusional disorders**	58,953	9.8%	35,389	12.7%	23,564	7.3%
**F30-F39**	**Mood [affective] disorders**	167,234	27.8%	62,494	22.3%	104,740	32.6%
**F40-F48**	**Neurotic, stress-related and somatoform disorders**	122,508	20.4%	43,355	15.5%	79,153	24.6%
**F50-F59**	**Behavioral syndromes associated with physiological disturbances and physical factors**	15,929	2.7%	3204	1.1%	12,725	4.0%
**F60-F69**	**Disorders of adult personality and behavior**	90,293	15.0%	36,335	13.0%	53,958	16.8%
**F80-F89**	**Disorders of psychological development**	19,294	3.2%	14,999	5.4%	4295	1.3%
**F90-F98**	**Behavioral and emotional disorders with onset usually occurring in childhood and adolescence**	34,326	5.7%	20,447	7.3%	13,879	4.3%
**Subtotal**	**Subchapters in this table**	594,050	98.3%	275,164	98.4%	315,767	98.3%
**Total**	**Total number of patients treated in Dutch specialist psychiatric care**	601,045^a^	100%	279,709^a^	46.5%	321,336^a^	53.4%

Patients aged 18 through 75, treated in Dutch specialist psychiatric care in 2010. Diagnoses are broken down by ICD-10 blocks. Blocks that occurred less than 1% are not shown in the table. ^a^Totals include also ICD-10 blocks and disorders with an occurrence of less than 1% (F70-F79), mental retardation (*n* = 16); (F99-F99), unspecified mental disorder (*n* = 3119), and DBCs without a diagnosis (*n* = 6995)

In 2010, 217,887 (36.3%) of psychiatric healthcare utilizers also used medical care. Of all medical healthcare utilizers, 4.8% also used psychiatric healthcare. The following psychiatric disorder groups were found more often in HUMPCs compared to HUPCs: ‘organic mental disorders’ (POR 1.82), ‘behavioral syndromes associated with physiological disturbances and physical factors’ (POR 1.26), ‘mood [affective] disorders’ (POR 1.10), ‘neurotic, stress-related and somatoform disorders’ (POR 1.09), and ‘disorders of adult personality and behavior’ (POR 1.07). In contrast, ‘disorders of psychological development’ (POR 0.56) ‘schizophrenia, schizotypal and delusional disorders’ (POR 0.73), ‘behavioral and emotional disorders with usual onset occurring in childhood’ (POR 0.75), and ‘mental and behavioral disorders due to psychoactive substance abuse’ (POR 0.85) were found less frequently in HUMPCs compared to HUPCs (Table 3).

**Table 3:** Differences in healthcare utilization between HUMPCs and HUPCs

ICD code	Subchapter title	HUMPCs		HUPCs		Proportional occurrence	
		***n***	***%***	***n***	***%***	***POD***^a^	***POR***^b^
**Total**	**All patients with any mental and behavioral disorder diagnosis**	^c^217,887	36.30%	^c^383,158	58.80%	0.00%	mean 1.09
**Subtotal**	**Subtypes in this table**	213,985	98.20%	376,930	98.30%	−0.20%	mean 1.02
**F30-F39**	**Mood [affective] disorders**	64,415	29.60%	102,819	26.80%	2.70%	1.1
**F40-F48**	**Neurotic, stress-related and somatoform disorders**	46,911	21.50%	75,597	19.70%	1.80%	1.09
**F60-F69**	**Disorders of adult personality and behavior**	34,249	15.70%	56,044	14.60%	1.10%	1.07
**F10-F19**	**Mental and behavioral disorders due to psychoactive substance abuse**	22,126	10.20%	45,528	11.90%	−1.70%	0.85
**F20-F29**	**Schizophrenia, schizotypal and delusional disorders**	17,220	7.90%	41,733	10.90%	−3.00%	0.73
**F90-F98**	**Behavioral and emotional disorders with onset usually occurring in childhood and adolescence**	10,242	4.70%	24,084	6.30%	−1.60%	0.75
**F00-F09**	**Organic, including symptomatic, mental disorders**	7491	3.40%	7233	1.90%	1.60%	1.82
**F50-F59**	**Behavioral syndromes associated with physiological disturbances and physical factors**	6664	3.10%	9265	2.40%	0.60%	1.26
**F80-F89**	**Disorders of psychological development**	4667	2.10%	14,627	3.80%	−1.70%	0.56

Occurrence of psychiatric disorders in HUMPCs and HUPCs. Occurrences smaller than 1% were excluded. The table is sorted on occurrence of psychiatric ICD-blocks in the co-occurring medical service use group. Proportional occurrence is expressed as difference in percentage points (^a^Proportional Occurrence Difference, POD) and ratios (^b^Proportional Occurrence Ratio, POR). ^c^Totals include ICD-10 blocks and disorders with an occurrence of less than 1% including: F70-F79 mental retardation (*n* = 16, POD 0.0%, POR 1.76) and F99-F99 unspecified mental disorder (*n* = 3119, POD 0.0%, POR 0.95)

### Claims expenditures

In 2010, total claims expenditures in specialist psychiatric and medical healthcare were €4.53 billion and €10.71 billion, respectively. The total costs of claims for the HUMPCs (*n* = 217,887) accounted for €2.9 billion, equaling 19% of total claims in 2010.

Of all psychiatric claims in 2010, HUPCs (63.7%) claimed a total of €2.75 billion and HUMPCs (36.3%) accounted for €1.78 billion. Thus, HUMPCs claimed 65% of total psychiatric costs. Median psychiatric claims of HUMPCs were 10% higher overall compared to HUPCs. When medical healthcare was utilized, median psychiatric claims were higher in four sub-chapters: + 26% in substance use disorders, + 17% in disorders of psychological development, + 12% in mood disorders, and + 1% in disorders with onset usually occurring in childhood. In three sub-chapters, psychiatric claims were not affected by medical healthcare utilization (disorders associated with psychological disorders and physical factors, organic disorders, and neurotic, stress-related, and somatoform disorders). Finally, in two sub-chapters, psychiatric claims were lower in HUMPCs than HUPCs: − 22% in personality disorders and − 8% in schizophrenia spectrum disorders, respectively (see Table 4).

**Table 4:** Differences in psychiatric costs of claims between HUMPCs and HUPCs

ICD code	Subchapter title	HUMPCs		HUPCs		Proportional claims expenditures		
		***Median claims (€)***	***+/− IQR***	***Median claims (€)***	***+/− IQR***	***PCED psychiatric***^a^	***PCED total***^a^	***PCEDR***^b^
**F80-F89**	**Disorders of psychological development**	€ 2517	€ 4862	€ 2154	€ 4374	€ 363	€ 1023	17%
**F50-F59**	**Behavioral syndromes associated with physiological disturbances and physical factors**	€ 1925	€ 3172	€ 1925	€ 3076	€ -	€ 902	0%
**F00-F09**	**Organic, including symptomatic, mental disorders**	€ 1792	€ 2914	€ 1792	€ 2806	€ -	€ 2142	0%
**F90-F98**	**Behavioral and emotional disorders with onset usually occurring in childhood and adolescence**	€ 2002	€ 2850	€ 1987	€ 1856	€ 15	€ 646	1%
**F20-F29**	**Schizophrenia, schizotypal and delusional disorders**	€ 5117	€ 15,225	€ 5579	€ 12,182	€ -462	€ 217	−8%
**F10-F19**	**Mental and behavioral disorders due to psychoactive substance abuse**	€ 3030	€ 8833	€ 2411	€ 6697	€ 619	€ 1672	26%
**F60-F69**	**Disorders of adult personality and behavior**	€ 2339	€ 5138	€ 3011	€ 4944	€ -672	€ 123	−22%
**F40-F48**	**Neurotic, stress-related and somatoform disorders**	€ 1929	€ 3050	€ 1925	€ 2798	€ 4	€ 835	0%
**F30-F39**	**Mood [affective] disorders**	€ 2234	€ 3543	€ 1993	€ 3098	€ 241	€ 1054	12%
**Subtotal**	**Subchapters in this table**	€ 2346	€ 5074	€ 2140	€ 4604	€ 206	€ 979	10%

Median psychiatric claims of HUMPCs and HUPCs. Proportional declaration is expressed as difference in percentage points (^a^Proportional Claims Expenditures Difference, PCED) and ratios (^b^Proportional Claims Expenditures Ratio, PCEDR)

In 2010, HUMCs claimed €9.59 billion. With respect to HUMPCs, €1.12 billion was claimed for medical costs, 10.5% of total medical claims. The median medical claims of HUMPCs were 40% higher than for HUMCs. A total of 15 medical diagnostic groups showed higher medical claims with co-occurring psychiatric care utilization. The most marked differences in patients with co-occurring psychiatric disorders (see Table 5) were observed in the diagnostic groups ‘endocrine, nutritional and metabolic diseases’ (94%), ‘congenital malformations, deformation and chromosomal abnormalities’ (89%), and ‘diseases of the ear and mastoid process’ (86%).

**Table 5:** Differences in medical costs of claims between HUMPCs and HUMCs

ICD code	Chapter title	HUMPCs		HUMCs		Proportional declarations	
		***Median costs (€)***	***+/− IQR***	***Median costs (€)***	***+/− IQR***	***PDD (€)***^a^	
**IV**	**Endocrine, nutritional and metabolic diseases**	€ 390	€ 1695	€ 201	€ 654	€ 189	94%
**XII**	**Diseases of the skin and subcutaneous tissue**	€ 340	€ 693	€ 252	€ 471	€ 88	35%
**XVII**	**Congenital malformations, deformations and chromosomal abnormalities**	€ 397	€ 573	€ 210	€ 413	€ 187	89%
**XV**	**Pregnancy, childbirth and the puerperium**	€ 334	€ 366	€ 268	€ 240	€ 66	25%
**IX**	**Diseases of the circulatory system**	€ 527	€ 3598	€ 386	€ 830	€ 141	37%
**III**	**Diseases of the blood and blood-forming organs and certain disorders involving the immune mechanism**	€ 399	€ 841	€ 376	€ 599	€ 23	6%
**VI**	**Diseases of the nervous system**	€ 365	€ 418	€ 327	€ 355	€ 38	12%
**X**	**Diseases of the respiratory system**	€ 584	€ 822	€ 571	€ 614	€ 13	2%
**VIII**	**Diseases of the ear and mastoid process**	€ 240	€ 449	€ 129	€ 257	€ 111	86%
**XIV**	**Diseases of the genitourinary system**	€ 340	€ 632	€ 339	€ 407	€ 1	0%
**XI**	**Diseases of the digestive system**	€ 599	€ 1493	€ 486	€ 905	€ 113	23%
**XIII**	**Diseases of the musculoskeletal system and connective tissue**	€ 535	€ 1002	€ 441	€ 600	€ 94	21%
	Other *	€ 599	€ 1438	€ 673	€ 1145	-€ 74	−11%
**III**	**Neoplasms**	€ 544	€ 1356	€ 510	€ 1262	€ 34	7%
**XVIII**	**Symptoms, signs and abnormal clinical and laboratory findings not elsewhere classified**	€ 432	€ 815	€ 386	€ 556	€ 46	12%
**XIX**	**Injury, poisoning and other certain other consequences of external causes**	€ 484	€ 1209	€ 380	€ 604	€ 104	27%
**VII**	**Diseases of the eye and adnexa**	€ 356	€ 644	€ 304	€ 437	€ 52	17%
**Subtotal**	**Chapters in this table**	€ 723	€ 1913	€ 516	€ 1094	€ 207	40%

Median medical costs of HUMPCs and HUMCs. Proportional declaration is expressed as difference in percentage points (^a^Proportional Declaration Difference, PDD)

*The category other contains ICD code chapters I, XVI and XX

## Discussion

In this study of the 2010 nationwide medical and psychiatric hospital care claims data in the Netherlands, 217,887 individuals (4.4% of total claims) utilized both medical and psychiatric hospital care. Compared with HUPCs, HUMPCs were more often diagnosed with: organic mental disorders behavioral syndromes associated with physiological disturbances and physical factors; mood [affective] disorders; neurotic, stress-related, and somatoform disorders; and disorders of adult personality and behavior. Claims for these HUMPCs accounted for €2.9 billion, equaling 19% of total claims in 2010. Moreover, the medical claims of HUMPCs were 40% higher than claims for HUMCs, and the psychiatric claims for HUMPCs were 10% higher than for HUPCs.

### Medical healthcare utilization of psychiatric patients

Medical healthcare utilization differences for psychiatric patients might be related to patient and health system factors [13]. In this subsection we concentrate on our findings with respect to these two (sets of) factors.

#### Patient factors

In respect of psychiatric patients, risk factors for medical illness and associated healthcare utilization are expected to be related to patient factors, such as socio-economic factors, medical factors such as the presence of severe mental illness (SMI) and comorbid substance use disorder, and other behavioral risk factors such as a lack of physical activity and high body-mass [27, 28]. There was an over-representation of HUMPCs compared to HUPCs within the sub-chapter personality disorders, known for the association with chronic medical conditions such as obesity, pain disorders, syncope, seizures and arthritis [29, 30]. In addition the sub-chapter ‘behavioral syndromes associated with physiological disturbances and physical factors’ was over-represented with respect to HUMPCs. Within this sub-group, for instance, eating disorders were associated with increased risks for serious medical illnesses and premature death [31].

The presence of mental illness may in some cases reduce somatic care-seeking. For example, people with mental illness may have difficulties engaging in health services, reporting medical problems, and distinguishing physical symptoms from the symptoms of mental illness, especially if health services were non-inclusive or perceived to be non-inclusive [13]. These mechanisms might then lead to reduced healthcare utilization. The finding that psychotic spectrum disorders were under-represented in HUMPCs compared with HUPCs appeared to be compatible with our hypothesis and previous work [32]. For example, patients with anxiety disorders or schizophrenia were less frequently diagnosed with hypertension than the general population. Additionally, patients with schizophrenia less frequently used antihypertensives and lipid-lowering drugs [32]. Swildens et al. showed that Dutch patients with non-affective psychotic disorders were prescribed somatic medication less frequently and experienced lower somatic healthcare utilization [17].

#### Health system factors

Differences in healthcare availability and quality might contribute to psychiatric patients’ poor physical health outcome [13]. These differences include an inferior standard of care, the wrongful attribution of medical symptoms to psychiatric conditions (‘diagnostic overshadowing’), and clinicians’ reluctance to provide specific medical procedures due to perceived intolerance to psychological stress, difficulty getting informed consent, compliance issues, or substance misuse [33, 34]. Such mechanisms might have contributed to our finding that disorders of psychological development (including language disorders and speech disorders) were under-represented in HUMPCs.

Health services are often perceived as non-inclusive by people with psychiatric illnesses. Fragmentation of care and social stigma further compromise adequate access to healthcare for these groups [14, 35]. Our data, in all likelihood, reflected the under-representation of patients with schizophrenia and substance use disorders in medical health care since these groups were under-represented in HUMPCs compared to HUPCs.

### Claims expenditures

Inadequate healthcare utilization by psychiatric patients may result in higher claims expenditures of HUMPCs, as shown in our study. These patients have a higher risk for medical illness and experience more extended lengths of hospital stay. Restrictions in accessibility (as described above) and delayed diagnosis and treatment might lead to avoidable ED visits and (re) admissions [5]. LOS and readmissions were considered important cost-drivers of hospital care [36] and might thus explain many of the excess claims that we found for HUMPs compared to HUPCs and HUMCs.

In contrast, diagnoses of personality disorders and psychotic spectrum disorders were associated with lower psychiatric claims in HUMPCs than HUPCs. Personality disorders are known to pose a high economic burden [37]. Thus, it is remarkable that we found lower median costs of psychiatric claims for HUMPCs than HUPCs in these patient groups. A possible explanation is the lower probability of admission to specialized care for patients with personality disorders found by van Veen et al. [38]. A possible reason for the lower number of psychiatric claims for psychotic spectrum disorders in HUMPCs compared to HUPCs was that these patients tended to avoid healthcare, presenting first in medical hospitals and only when they had developed acute medical symptoms, as found by Swildens et al. [17]. Future research should examine how care can be effectively improved to alleviate this situation.

### Public health implications

Our study found that HUMPCs, in almost all subgroups, had increased healthcare costs compared to HUMCs and HUPCs. Because patients with a mental disorder are more likely to have a physical disorder, it is important that physical disorders are detected early. The efforts to improve physical health in mentally ill patients could be to focused on patients with severe mental disorders [39]. Our study shows that a focus on public health is important for all patients with mental health problems. This should take into account, social determinants – the conditions in which people are born, grow up, live, work and age – and unhealthy lifestyles are intertwined and negatively affect the risk for physical and psychiatric disorders [13]. It is important to intervene early in life on social and lifestyle risk factors that significantly reduce the risk of physical and psychiatric illness disorders [13]. In the long run, hospital healthcare utilization may decrease due to these efforts.

### Limitations

In the Netherlands health care insurance is mandatory, thus replication of our results will not easily be accomplished in countries with different accessibility of care. On the other hand, the results will most likely be replicable for citizens with an inclusive health insurance package. A further limitation may be that this nationwide data was 10 years old, but since no major reforms have taken place, it can be assumed that general healthcare utilization patterns were likely to have been stable over time. It is reassuring that our findings are in line with those reported in recent literature. Hence, we consider these results to be representative both currently and for other countries with inclusive health insurance packages. A significant finding was that median claims in specialist medical care were considerably lower than median claims in psychiatric hospital care. This can be explained by a greater emphasis on outpatient care within specialist medical care. Many outpatients’ DBCs have low reimbursement rates, and thus median claims for medical care are lower than for psychiatric hospital care. In this nationwide study, we used POD and POR. Other studies that examined the impact of concurrent medical and psychiatric disorders on health utilization used odds ratios (OR) [9–12] or latent class analyses [27, 28]. The calculation of the POR is comparable to that of the OR, and our results are therefore comparable to other studies. We only carefully speculated about possible patient and health system mechanisms that may explain the differences in healthcare utilization and claims in this study, however, our data were insufficient to formally examine these interpretations.

## Conclusion

In this representative nationwide dataset, marked medical healthcare utilization differences were shown when comparing psychiatric disorder groups. These differences might be related to patient and health system factors. HUMPCs displayed 40% higher median medical costs claims than HUMCs. In addition, HUMPCs psychiatric claims were 10% higher than for HUPCs. Medical and psychiatric claims of HUMPCs appeared higher for all sub-groups, except for patients with personality and psychotic disorders who were associated with lower psychiatric claims. To improve the accessibility and quality of healthcare and to prevent further morbidity and mortality in patients with co-occurring medical and psychiatric hospital care utilization, policymakers and healthcare providers should acknowledge the frequent co-occurrence of medical and psychiatric disorders and its impacts.

## Data Availability

The data that support the findings of this study are available from the Dutch Health Authority, but restrictions apply to the availability of these data, which were used under license for the current study, and so are not publicly available. Data are however available from the authors upon reasonable request and with permission of the Dutch Health Authority.
